# Rare Presentation of Sinus of Valsalva Aneurysm Treated by Aortic Valve Reimplantation Technique

**DOI:** 10.21470/1678-9741-2020-0672

**Published:** 2022

**Authors:** Johannes Petersen, Theresa Holst, Simon Pecha, Hermann Reichenspurner, Evaldas Girdauskas

**Affiliations:** 1 Department of Cardiovascular Surgery, University Heart & Vascular Center Hamburg, Hamburg, Germany.

**Keywords:** Sinus of Valsalva, Aortic Aneurysm, Heart Atria, Heart Failure, Replantation

## Abstract

Sinus of Valsalva aneurysm is a rare cardiac abnormality which can be acquired or of congenital origin. A spontaneous rupture into the right atrium is possible and, if not adequately treated, may result in a progressive heart failure due to the left-to-right intracardiac shunt. If ruptured sinus of Valsalva aneurysm is diagnosed, surgical repair is indicated, and different surgical techniques have been reported. If concomitant aortic regurgitation is present, aortic valve replacement is usually performed. Herein, we describe an uncommon clinical presentation of a ruptured sinus of Valsalva aneurysm which has been corrected by aortic valve reimplantation.

**Table:** Abbreviations, acronyms & symbols

CT	= Computed tomography
TEE	= Transesophageal echocardiography

## INTRODUCTION

Sinus of Valsalva aneurysm is a rare cardiac abnormality which can be of congenital origin (*e.g*., connective tissue disorder) or acquired, caused by an inflammatory disease (*e.g*., syphilis, endocarditis)^[1]^. Most commonly, the aneurysm is located either in the right or the non-coronary sinus while the left coronary sinus is rarely involved^[1]^. During the time course, a spontaneous rupture of sinus of Valsalva aneurysm into the right atrium is possible and, if not adequately treated, may result in a progressive heart failure due to the left-to-right intracardiac shunt or even in a sudden cardiac death^[1]^. Depending on the size of perforation, clinical presentation may vary between an asymptomatic heart murmur, mild dyspnea, chest pain^[2]^, or even signs of acute heart failure^[1]^. Diagnosis is confirmed with transesophageal echocardiography (TEE), contrast-enhanced computed tomography (CT), or magnetic resonance imaging^[3]^. If ruptured sinus of Valsalva aneurysm is diagnosed, surgical repair is indicated and different surgical techniques have been reported, depending on the type of the aneurysm^[4]^. If concomitant aortic regurgitation is present, aortic valve replacement is usually performed^[5]^. Herein, we describe an uncommon clinical presentation of a ruptured sinus of Valsalva aneurysm which has been corrected by means of aortic valve reimplantation and simultaneous aortic valve repair.

## TECHNIQUE

**Fig. 1:**
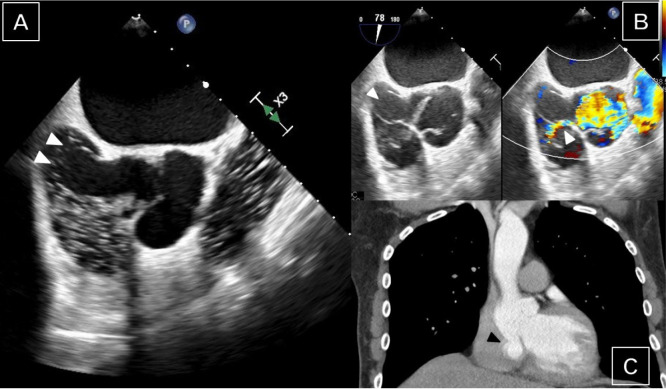
Preoperative transesophageal echocardiography (A, B) and contrast-enhanced computed tomography imaging (C) showing the sinus of Valsalva aneurysm with an aorto-right atrial shunt (arrows).

**Fig. 2:**
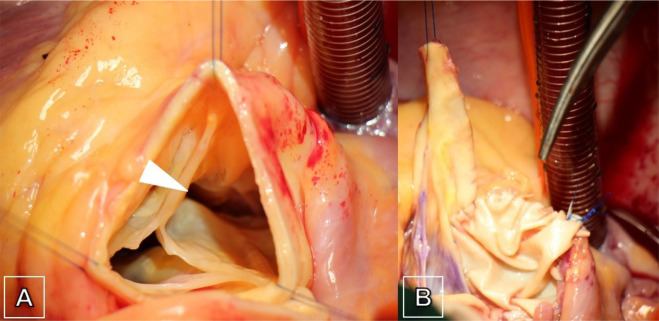
Intraoperative findings of the perforated sinus of Valsalva aneurysm with an aorto-right atrial shunt (arrow) and the asymmetric tricuspid aortic valve configuration (A) and the closure of the right atrial shunt with bovine pericardial patch (B) before aortic valve reimplantation was performed.

A 57-year-old male patient was referred to our hospital for catheter-based ablation due to paroxysmal atrial fibrillation. The patient reported intermittent palpitations, dizziness, and a reduced quality of life. Otherwise, the patient was healthy and had no previously diagnosed connective tissue disorder. During the preprocedural diagnostic work-up, a TEE was performed and revealed an ascending aortic aneurysm of 43 mm and a concomitant sinus of Valsalva aneurysm of 20 × 20 mm protruding into the right atrium (Figure 1A) associated with an aorto-right atrial shunt (Figure 1B). Right atrial dimensions were significantly enlarged. Mild aortic valve regurgitation was present and left ventricular systolic function was preserved. Subsequent cardiac CT scan confirmed the diagnosis of perforated sinus of Valsalva aneurysm with contrast medium shunting into the right atrium (Figure 1C). Intraoperatively, ruptured sinus of Valsalva aneurysm originating from the non-coronary and the right coronary sinus was present (Figure 2A). Native tricuspid aortic valve was highly asymmetric and right coronary cusp showed geometric height of only 15 mm in combination with a reduced effective height of 4 mm. (Figure 2A). The geometric height of the non- and the left coronary cusp was 22 mm and 18 mm, respectively. Despite this complex asymmetric anatomy, a valve-sparing procedure was planned, taking into account a good quality of the native cusps. First, the aorto-right atrial shunt was closed, and the right atrial roof restored using a bovine pericardial patch (40 × 20 mm) (Figure 2B). Next, aortic sinus tissue was completely resected preserving only both coronary buttons. During the reimplantation, the three aortic valve commissures were asymmetrically attached into the 28-mm Gelweave^TM^ Valsalva Graft to mimic the original valve orientation and to achieve sufficient coaptation of aortic cusps. In addition, central plication suture of the right coronary cusp was performed to reach the effective cusp height of 8 mm. Furthermore, pulmonary vein isolation was performed with bipolar radiofrequency and the left atrial appendage was closed using an AtriClip® LAA Exclusion System. After weaning from cardiopulmonary bypass, the TEE showed a normal aortic valve function with only trace residual aortic regurgitation, no residual left-to-right cardiac shunt, and complete closure of the left atrial appendage. The patient recovered uneventfully after the surgery and was discharged in stable sinus rhythm and only trace aortic regurgitation with a mean transvalvular gradient of 13 mmHg.

## DISCUSSION

This case represents an aortic valve sparing root procedure which aims to maintain the geometry of aortic valve considering the marked asymmetry of aortic root. The challenge of reimplantation procedure in this scenario is to keep the asymmetric configuration of aortic root due to extremely dilated non-coronary sinus and large non-coronary cusp of the aortic valve. Aortic valve annulus was almost non-existent below the perforated sinus of Valsalva aneurysm in the non-coronary sinus and was therefore fixed from inside to outside using the standard pledgeted subannular anchoring sutures. In this specific case, the defect was in the right atrial groove well above the tricuspid valve plane. Therefore, the right atrium was not separately opened.

## CONCLUSION

Despite an uncommon clinical presentation, a ruptured sinus of Valsalva aneurysm can be detected by comprehensive preoperative work-up. Further, an aortic valve reimplantation is possible even in a complex asymmetric anatomy if the geometry of the aortic valve is respected.
